# MLA Colleague Connection: a transition to a virtual mentoring program

**DOI:** 10.5195/jmla.2022.1356

**Published:** 2022-10-01

**Authors:** Nell Aronoff, Heather S. Healy, Emily J. Glenn

**Affiliations:** 1 naronoff@buffalo.edu, Associate Librarian, University Libraries, University at Buffalo, Buffalo, NY.; 2 heather-healy@uiowa.edu, Clinical Education Librarian, Hardin Library for the Health Sciences, University of Iowa, Iowa City, IA.; 3 emily.glenn@unmc.edu, Interim Dean, Leon S. McGoogan Health Sciences Library, University of Nebraska Medical Center, Omaha, NE.

**Keywords:** Mentors, mentoring, mentorship, professional organizations, Medical Library Association, medical librarians

## Abstract

**Background:**

Since 2003, the MLA Membership Committee has facilitated an in-person mentoring program called Colleague Connection at the annual meeting. The program hinged on meeting attendance, so members who could not attend were excluded. The 2020 virtual meeting created an opportunity to rethink the Colleague Connection experience. Three members of the Membership Committee developed an expanded and virtual version of the mentoring program.

**Case Presentation:**

Colleague Connection was promoted via the MLA ’20 vConference Welcome Event, MLAConnect, and email lists. The 134 participants were matched based on same-chapter preference, library type, practice area interest, and years of experience. Mentees chose mentor-mentee or peer pairs, resulting in 4 peer matchings and 65 mentor-mentee matchings. Pairs were encouraged to meet monthly, and conversation prompts were provided. A Wrap-Up Event was held for participants to talk about their experiences and network. A survey evaluated the program and sought suggestions for improvement.

**Conclusion:**

The online format boosted participation, and the format change was well received. In the future, a formal orientation meeting and communication plan can ensure pairs make their initial connections and provide clarity about program details, expectations, timelines, and contact information. The type of pairings and size of the program are important considerations for the feasibility and sustainability of a virtual mentoring program.

## BACKGROUND

Mentoring programs are a common feature of professional library organizations. Organizations such as the Australian Library and Information Association (ALIA) [1, 2], the Chartered Institute of Library and Information Professionals (CILIP) [3, 4], the Association of Research Libraries (ARL) [5], and multiple divisions and round tables of the American Library Association (ALA) [6–11] all offer their members mentoring programs. Though more specifically focused on leadership, the Canadian Health Libraries Association (CHLA/ABSC) provides a mentoring program for developing library leaders [12]. Similarly, the Association of Academic Health Sciences Libraries (AAHSL), in conjunction with the National Library of Medicine (NLM), runs the NLM/AAHSL Leadership Fellows Program focused on developing health sciences library directors [13]. The Network of the National Library of Medicine (NNLM) has also hosted mentorship programs [14].

A variety of mentoring opportunities have existed through the Medical Library Association (MLA). Past annual meetings have provided interactive mentoring sessions [15, 16]. Other meeting sessions have provided mentorship-related content, including the importance of mentorship [17], activities by colleagues in a local area [18], individual efforts at an institution [19], and self-mentoring to supplement formal programs [20]. Some MLA chapters and caucuses (née sections) have created their own mentoring programs [21–23]. Since 2010, MLA has provided year-long mentorship through the Rising Stars program; however, the program is limited to only a few members each year [24, 25].

The Colleague Connection initiative began in 2003 as a mentoring program for first time MLA annual meeting attendees. Led by the MLA Membership Committee with support from MLA staff, the goals of Colleague Connection are to strengthen relationships between MLA members and promote awareness of opportunities to grow within the organization. By introducing new members to the MLA structure and possibilities for involvement with groups or service roles, the Colleague Connection event, as the only MLA-wide mentoring program, supports retention and development of a “professional home” for MLA members. In past years of the program, a first-time MLA annual meeting attendee was paired with a more experienced attendee. The connection focused on the one-time, in-person meeting and helped new attendees navigate the meeting. Since the connection hinged on annual meeting attendance, members who could not attend the meeting were excluded. The mentoring relationships were generally short-lived and were limited to the parameters of what mentors' and mentees' conference schedules allowed.

In the spring of 2020, as MLA and other organizations began to shift to online-only programming due to the COVID-19 pandemic, we (three MLA Membership Committee members) decided to plan an extended online Colleague Connection program. Several articles and meeting abstracts detail the challenges and benefits of developing online mentoring programs through professional organizations [2, 4, 21, 25–27]. Eldredge similarly describes challenges and opportunities discovered while providing evidence-based practice online mentoring for fellow librarians [28]. Many of these reports were individual or small pilot programs limited to a small number of applicants. This case report expands upon that literature by detailing our experience with an online, organization-wide mentoring program.

## CASE PRESENTATION

### Planning and Promotion

As MLA and other organizations moved programming online, the MLA Membership Committee made quick plans to offer an online mentoring program. We held a planning meeting in mid-July 2020 to devise the overall structure, map out a timeline, and assign responsibilities for recruitment, matching, and communicating with participants.

The new virtual Colleague Connection program was announced on July 27, 2020, during the MLA vConference Welcome Event. To reach members who did not attend the Welcome Event, weekly MLAConnect emails advertised the program from late July through mid-September. Colleague Connection was also promoted via the MLA Community Council and through chapter email lists.

We marketed the program as a way for both new and experienced members to expand their professional networks. Mentors were also told that mentoring a colleague would help someone navigate a new organization and profession. The program dates were advertised as October 2020 to January 2021. Anyone interested in participating in the program signed up via an online form on the MLA website.

#### Participants

One hundred thirty-four people signed up, including MLA members from the United States, Canada, and 3 other countries. This represents approximately 5.4% of MLA's total membership of 2,469, as reported in the 2020-2021 MLA Headquarters Annual Report [29]. Seventy-four mentees and 60 mentors signed up.

Mentees could choose to be matched with either a fellow new member or a mentor. Participants provided their name, email address, institutional affiliation, geographic location, years in librarianship, librarian type, chapter affiliation, preference for a same-chapter match, and primary interest by practice area. We also asked mentors if they would be willing to mentor more than one person.

Most participants came from academic libraries, followed by hospital libraries. Mentees averaged six years of experience. Fifty-seven percent of mentees (n=42/74) had 1 to 3 years of experience. Mentors averaged 15 years of experience, and 70% of mentors (n=42/60) had more than 10 years of experience in librarianship. Sixteen mentees and 6 mentors wanted to be matched with someone from their own region or chapter, but most did not express a preference. Education (n=70/134, 52%) and Information Services (n=64/134, 48%) were the two most frequently selected areas of practice.

Matching was based on preference for same-chapter match, type of library served, primary interest by practice area, and years of experience. Once completed, sixty-five mentees were matched with a mentor. Since there were more mentees than mentors, five mentors were assigned two mentees. Nine mentees opted to connect with a peer, forming three pairs and one trio.

#### Program

All participants were notified of their matches by early October 2020 and were encouraged to meet for the first time that month. It was up to the pairs to establish their own meeting times and frequency and to determine the meeting format. We hoped pairs would meet at least once a month during the four-month program.

Email messages sent at the beginning of each month provided some structure to the program and served as a reminder to meet. The initial email shared some conversation prompts to help get things started. Prompts included questions like “How did you get where you are career-wise?,” “How are you involved in MLA?,” and “What do you wish you had known earlier in your career?” November's email highlighted volunteering to serve on a jury or committee, joining a caucus, presenting at the annual meeting, or applying for the MLA Rising Stars program. In December, we included a recap of questions from the October email. In January, we suggested pairs discuss how they keep up with the literature and how they create and maintain CVs and resumes. We also promoted a Wrap-Up Event and asked for volunteers to share their experiences from the program.

The Wrap-Up Event, held on January 28, 2021, marked the official end of the program. More than forty people attended the forty-five-minute event. The MLA Membership Committee Chair and the MLA President each gave introductory remarks. Three mentorship pairs talked about their experiences with the program before we offered two rounds of small group networking in breakout rooms. Pairs expressed that they were glad to get the chance to connect with another MLA member. Other attendees mentioned in the chat that they enjoyed the program.

#### Survey

In early February 2021, we surveyed participants about their experience in the virtual Colleague Connection program (Appendix A.) The survey was open for one week, from February 4 to February 11, 2021. The online survey was generated using Qualtrics through the University of Iowa. The University of Iowa Human Subjects Office determined the survey did meet the regulatory definition of human subjects research and therefore did not require IRB review. After an introductory question about how the survey respondent participated in Colleague Connection, there were two versions of the survey—one for mentors and one for mentees and peer pairs. After the survey closed, results were exported to Microsoft Excel. Qualtrics provided minimums, maximums, means, standard deviations, variances, count data, and percentages. In some cases, we combined the responses for mentors and mentees. Responses to the open-ended question were grouped thematically.

The survey asked about the number of times participants met and if that was sufficient, whether they planned to continue meeting, if the frequency of communications was sufficient, if mentors would participate again, if mentees felt the program was beneficial, and if mentees would recommend it to another new member. Fifty-four percent of participants (n=73/134) completed the survey, including 62% of mentors (n=37/60) and 49% of mentees (n=36/74).

Most participants reported meeting 3 to 4 times during the 4-month program. During that time, the mean for mentors was 3.5 meetings and the mean for mentees was 3.8 meetings. More than half of the survey respondents (n=41/73, 56%) indicated that they would continue to meet with their mentor/mentee after the formal program concluded, while 29% (n=21/73) said they might meet, and 15% (n=11/73) reported that they did not plan to meet in the future.

Seventy-four percent of respondents (n=54/73) indicated the 4-month program was an appropriate length of time. Fourteen percent of mentors (n=5/37) and a third of the mentees (n=12/36) said the program was too short. None of the mentors thought the program was too long, but two mentees (n=2/36, 6%) would have preferred a shorter program duration.

When asked whether the communication frequency was sufficient, 90% of respondents (n=66/73) indicated the 1 email per month from coordinators was sufficient. Five people (7%) indicated the question was “not applicable” to them, and 2 (3%) answered “no.” Unfortunately, the peer pairs were inadvertently left off the email lists. These peer groups received an initial email with their matches; however, they did not receive any further communication from us. Respondents who answered “no” to this question were mentees and not members of peer pairs.

Since the program was designed to support new members, we asked the mentees if the number of times they met was sufficient and whether the program was beneficial to them. Three-quarters of mentees (n=27/36) felt the number of times they met with their mentor or fellow new member was sufficient. Concerning the program's benefit, 31 mentees (n=31/36, 86%) indicated the program was “definitely” or “mostly” beneficial.

Most mentors (n=29/37, 78%) replied that they would participate in the program again, while the rest (n=8/37, 22%) said “maybe.” Similarly, we were curious whether the mentees would recommend the program to other new MLA members. Most mentees (n=31/36, 86%) selected “yes.”

At the end of the survey, we asked an open-ended question to determine how the program could be improved. Thirty-six respondents provided additional feedback. The following themes emerged: expressions of thanks; comments about communication; suggestions and observations about the program format, frequency, length, and timing; examples of pairings that were not as successful; cases of scheduling difficulties; and explanations of program benefits.

Regarding program format, frequency, length, and timing, a couple of mentors commented that they were usually very busy during the annual meeting and appreciated that this more flexible schedule allowed them to spend more time getting to know their mentee. Three mentees felt an introductory meeting would improve their experience. One person offered that the program could run year-round, while another felt that offering it twice a year would allow more people to participate. Four respondents suggested the program should be longer than four months. One person proposed the timing could overlap with the annual conference to give participants the opportunity to meet beforehand and discuss their experiences at the annual conference afterwards.

Communication was another theme that surfaced in the open-ended comments. One person mentioned sending an email after the initial pairing to make sure everyone successfully connected with their mentor or fellow new member. In addition, four people commented they did not receive the monthly emails.

In terms of reception, ten respondents provided notes of thanks or mentioned that they thought the new version of Colleague Connection went well. Four mentees highlighted program benefits. For example, a recent MLS graduate explained that their mentor helped them become “a better medical librarian,” gave them resume advice, and prompted them to join a caucus.

## DISCUSSION

Due to the COVID-19 pandemic, we moved the Colleague Connection program online. We recognized that moving to an online format provided an opportunity to improve and expand the program. Since the event previously occurred during the in-person MLA annual meeting, most pairs only had time to meet once, given the limited meeting time and competing demands on their attention. Aware of these limitations, we hoped to offer an online mentoring experience that met the program goals and offered more flexibility to participants to decide when and how often to meet. We also sought to help foster connection between MLA members several months into a stressful global pandemic.

We attribute the success of the 2020 Colleague Connection event to several factors.

- The virtual format enabled more people to participate since the program was open to all MLA members and participation was not tied to annual meeting attendance. In 2019, there were 66 participants in the in-person program, and the virtual version in 2020 saw the number of participants double to 134.
- The event was spread over four months, allowing participants more time for discussion and the opportunity to meet more than once. We hoped that participants would meet once a month, and most survey respondents indicated they met three to four times.
- The timing of the mentoring session did not overlap with the annual meeting, allowing mentors and mentees to discuss meeting participation ahead of the May 2021 virtual conference. One conversation prompt encouraged participation in the meeting by preparing an abstract for submission. During the Wrap-Up Event, we heard of an instance where a mentor encouraged a mentee to submit an abstract for MLA.

Although the first iteration of the new Colleague Connection program went well, we know it could be better. Potential improvements center on orientation, communication, and value. The kickoff was all done via email: we did not offer orientation meetings. In hindsight, we could have held a virtual meeting at the start so all participants could mingle and meet their mentors or mentees for the first time. At the Wrap-Up Event, some participants mentioned they had not participated in a professional mentoring program before and may not have known what to expect. Outlining basic expectations, the timeline, and program communication could have helped people feel ready to participate sooner and more fully.

An orientation would also help ensure pairs met at least once. Not all participants found the time to meet due to busy schedules and different time zones. In addition, a few matches never connected, which was evident in some of the survey responses and in our experience hearing from participants during the program. Hines reported similar issues, noting in that program that a survey respondent explained that she “never received the contact info for her protégé” but neither party reached out to organizers for help [27]. In future iterations, a better communication plan could address these situations by having a contact person and action plan for matches that do not work out.

This case study provides a window into one online mentoring program, but it has some limitations. Half of the 2020 Colleague Connection participants did not fill out the survey, so we do not know about their experience. We do not have historical information about participants or outcomes of the in-person Colleague Connection events. Participants were not specifically asked about the relationship they developed with their matches and whether their participation in the program led to more engagement; however, we received some feedback about this during the live Wrap-Up Event.

Although participants were asked whether they would continue to meet after the official program ended, they were not asked to elaborate on their responses. Asking this question could have given some additional insight into the connections. Additionally, we did not set out to discover or follow known best practices as we transitioned from in-person to online. Like many other groups, we quickly pivoted and drew on our collective experiences with the Colleague Connection program to provide the best program under the circumstances.

We suggest future post-event survey questions could offer choices of specific potential benefits of the program and goals of the MLA Membership Committee, such as more engagement in the organization or a higher comfort level with certain aspects of their work.

Despite this, a few open-ended responses yielded important information about program benefits. Respondents also offered constructive feedback about how to strengthen the program. Hines reported a similar experience with their survey, noting “the open-response questions were the most valuable and informative” [27]. Intentional reflection and self-assessment about participants' performance in the program could be meaningful [28].

We offer the following recommendations for future events or others planning similar virtual mentoring programs for their professional organization. The first three recommendations build on survey respondents' answers to the open-ended question that asked them how future iterations of Colleague Connection could be improved.

- Hold an online orientation at the outset of the program.
- In-person events at annual meetings should correspond with a virtual component before or after the in-person meeting.
- Consistent communication is important. Participants should be informed about program details and expectations, the timeline, and how to reach organizers if they encounter any issues. Along the same lines, Hines proposed that feedback should be solicited “at a halfway point through the program to catch problems … before they become big issues later” [27].
- A discussion board could broaden and deepen the experience and allow for more connections. Ma and Wong and Hines describe this communication piece as a potential supplement to meetings among the pairs [26, 27].
- Limit pairings to mentor-mentee pairings only, reserving peer-to-peer mentoring for another program. More experienced mentors have more insight into the organization, and since one objective of Colleague Connection is to increase engagement in MLA, a mentee-mentor match is best in this context. The single option also simplifies coordination for organizers.
- Consider matching based on areas of need or interest—for example, job and resume support; solo librarianship; tenure-track positions; or liaison-specific roles.

Other MLA groups offer dedicated mentoring programs. MLA's Rising Stars program, which accepts four mentees per year, is currently the most supported. Chapter and caucus-specific mentoring efforts are not standard across groups or years; hence, the MLA Membership Committee's Colleague Connection is the longest running, all-MLA mentorship offering. Other MLA groups might be able to match participants by identity or background more than our MLA-wide initiative and could offer peer-to-peer connections.

In this initiative, the Colleague Connection program could be sustained in a virtual or hybrid format with continued leadership from the MLA Membership Committee and MLA staff. The virtual program was coordinated by three MLA Membership Committee members; one MLA staff member coordinated the virtual program. If Colleague Connection program demand increases or the scope of the mentoring offered by the Membership Committee grows, program support will need to increase. Some survey respondents suggested lengthening the program or offering it more than once during the year; however, the small number of people serving on the Membership Committee would need assistance from other groups to implement those changes.

Overall, participants who completed our survey were pleased with the virtual Colleague Connection program. Though necessitated by the pandemic, it brought about positive changes to a long-running Membership Committee initiative.

## Data Availability

Data about Colleague Connection participants cannot be made publicly available because they contain personally identifiable information. Survey data associated with this article are available in the University at Buffalo Institutional Repository (UBIR) at http://hdl.handle.net/10477/82987.

## SUPPLEMENTAL FILES

**Appendix A:** MLA Colleague Connection Survey
