# Supplementary material for: MLA Colleague Connection: a transition to a virtual mentoring program
Source: J Med Libr Assoc. 2022 Oct 1;110(4):513–9. doi: 10.5195/jmla.2022.1356 (PMC10124597; doi:10.5195/jmla.2022.1356)
Supplement: Supplementary file 1 — Appendix A: MLA Colleague Connection Survey [file jmla-110-4-513-s01.pdf]

Appendix A: MLA Colleague Connection Survey

Survey Instrument

Thank you for participating in the first virtual Colleague Connection program. This survey should take only a few minutes to complete and will help the MLA Membership Committee improve the program in the future. This survey received IRB exemption from the University of XXXX Medical Center.

Question for All Participants

Q1 - Select how you participated in Colleague Connection.
- Mentor
- Mentee
- Member of peer pair

Question Branch for Mentors

Q2 - How many times did you meet with your mentee over the past four months?
- 0 times
- 1 time
- 2 times
- 3 times
- 4 times
- 5 or more times

Q3 - Indicate the appropriateness of the length of program (i.e., four months).
- The duration was too short
- The duration was just right
- The duration was too long

Q4 - Was the once per month communication from the organizers sufficient?
- Yes
- No

Q5 - Would you participate in this program again?
- Yes
- Maybe
- No

Q6 - Do you and your mentee plan to continue meeting now that the official program has concluded?
- Yes
- Maybe
- No

Q7 - How could this program be improved in the future?

Question Branch for Mentees/Peer Pairs

Q2 - How many times did you meet with your mentor or fellow new member over the past four months?

- 0 times
- 1 time
- 2 times
- 3 times
- 4 times
- 5 or more times

Q3 - Was the number of times you met sufficient?

- Yes
- Maybe
- No

Q4 - Indicate the appropriateness of the length of program (i.e., four months).

- The duration was too short
- The duration was just right
- The duration was too long

Q5 - Was the once per month communication from the organizers sufficient?

- Yes
- No
- Not applicable

Q6 - Did you find this program beneficial?

- Definitely yes
- Mostly yes
- Undecided
- Mostly no
- Definitely no

Q7 - Would you recommend this program to another new member of MLA?

- Yes
- Maybe
- No

Q8 - Do you and your mentor or fellow new member plan to continue meeting now that the official program has concluded?

- Yes
- Maybe
- No

Q9 - How could this program be improved in the future?
